# The Influence of Community-Based Integrated Care on the Physical and Mental Health of Older Adults: Evidence from China

**DOI:** 10.5334/ijic.9099

**Published:** 2026-09-30

**Authors:** Qinglin Xu, Cangcang Jia

**Affiliations:** 1 School of Economics and Management, Anhui University of Chinese Medicine, 350 Longzihu Road, Hefei 230012, China; 2 School of Health Policy & Management, Nanjing Medical University, 101 Longmian Avenue, Jiangning District, Nanjing 211166, China

**Keywords:** older adults, community-based integrated care, self-rated health, physical health, mental health

## Abstract

This study examined the association between community-based integrated care and older individuals’ self-rated health, physical health, and mental health. The core components of community-based integrated care services include four major components: daily care, medical services, health services and psychological support. The study used data from the 2018 Chinese Longitudinal Healthy Longevity Survey (CLHLS). The study employed a rigorous methodological framework to mitigate potential endogeneity biases, integrating ordinary least squares (OLS) regression with propensity score matching (PSM) and an instrumental variables (IV) approach. This multi-method design aimed to address both observed confounders and unobserved heterogeneity or reverse causality, thereby enhancing the robustness of causal inferences. The results of the study suggest that older persons who received community-based integrated care were associated with better self-rated health, physical health, and mental health than those who did not receive it. Overall, community-based integrated care was associated with greater health benefits among older persons aged 80 and above, women, older people living alone, and older people who reside in rural regions. Community-based integrated care is positively associated with the health status of older people, and a higher degree of community-based integrated care is associated with better physical and mental health among older people.

## Introduction

China has experienced a rapid increase in its aging population over the past decade, with individuals aged 65 and above comprising 14.9% of the total population by 2022 [1]. This demographic shift has led to emerging social risks associated with aging-related incapacity and insufficient care [2]. A shortage of geriatricians, specialist caregivers, and other essential human resources persists, hindering the development of a robust long-term care system in China [3].

The World Health Organization (WHO) highlights the necessity of developing long-term care systems to support older adults experiencing declining vitality and intrinsic capacity [4]. To address the increasing health risks and disease burden associated with aging, China’s National Health Commission outlined in the “14th Five-Year Plan for Healthy Aging” the goal of “promoting home- and community-based care services” [5]. The plan encourages collaboration between community health service centers and relevant institutions to enhance caregiving capabilities, including offering short-term and temporary care services for older individuals in their homes.

Older adults often prefer home and community-based care due to its benefits, such as maintaining interpersonal relationships, particularly through family ties, which foster security, intimacy, belonging, and autonomy [6]. Many countries have shifted focus from institutional care to community-based care, allowing older adults to remain in their families and communities while receiving adequate support [7,8]. By integrating available human resources and adapting to local contexts, community-based integrated care services can address the diverse needs of frail older adults [9]. This approach reduces the psychological distress caused by environmental changes, minimizes mobility limitations and social barriers, and lowers the consumption of medical resources [10,11].

The WHO defines integrated care for older adults as a person-centered, coordinated care model designed to optimize health service systems within the framework of healthy aging [12]. Its primary goal is to enhance the intrinsic and functional abilities of older individuals. Building on the concepts of “aging in place” [13] and “integrated care,” this study defines “community-based integrated care” as a family-centered, community-based, and professionally organized system that delivers home-based care for older adults. This model combines the familiarity of informal care with the expertise of institutional care, offering living assistance, health support, and emotional comfort to older individuals in their own homes.

While most research on community-based integrated care services focuses on policy design, service implementation [14], or factors influencing care demand [15,16], its health impact remains underexplored. From the perspective of social support theory, strong social support is critical for improving mental and physical health, offering protection during challenges and fostering positive emotional experiences [17]. Studies suggest that community-based home care services improve physical functioning and quality of life for older adults [18]. Moreover, the residence preferences of older individuals in China align with this care model [19], highlighting the significance of community-based support.

Receiving long-term care at home and within the community helps older adults reduce financial burdens, enabling them to allocate limited resources to daily living expenses. This improves their quality of life, satisfaction, and psychological well-being in later years [20]. Under the framework of welfare pluralism, integrated care emphasizes the consolidation and reorganization of care resources provided by families, society, and the market. Communities play a pivotal role in integrating these resources, making the development of community-based integrated care services essential for promoting the health and well-being of older adults who prefer to age at home [21].

This study utilizes data from the 2018 Chinese Longitudinal Healthy Longevity Survey (CLHLS) to empirically examine the influence of community-based integrated care services on the health of older adults, in an attempt to answer the following questions: Firstly, what is the impact of community-based integrated care services on the health of older adults? Secondly, are there any variations in the impact of community-based integrated care services on the health of older adults among different demographic groups?

## Methods

### Study Sample

This study uses data from the CLHLS, conducted by Peking University’s Center for Healthy Aging and Development and the National Development Research Institute (NDRI). Renowned for its reliability, validity, and robust representativeness, the CLHLS data have been widely recognized by academic communities both domestically and internationally [22,23]. The survey employed multi-stage stratified sampling, covering nearly half of the counties and cities in 23 provinces, municipalities, and autonomous regions in China, with follow-ups conducted from 1998 to 2018. It includes comprehensive data on socioeconomic status, community services, quality of life, and health issues. Across eight national surveys, 113,000 household interviews were conducted, with 67.4% of respondents aged 80 and above, a group in significant need of care.

This study uses 2018 CLHLS data to assess the association between community care and older individuals’ health. Respondents under 60 years old, as well as cases with missing, nonsensical, or incomplete responses, were excluded. The final sample includes 9998 older adults, with 396 receiving community-based integrated care and 9602 not receiving such services. The notable difference in sample size between the treatment group (n = 396) and the control group (n = 9,602) reflects the current stage of community-based integrated care development in China. Although the government has actively promoted home- and community-based care services, the simultaneous provision of all four service types, namely daily care, medical services, health services, and psychological support, remains limited to a relatively small proportion of communities, particularly in rural and less economically developed areas [3]. This low coverage rate is consistent with existing policy literature and national statistics on community care provision. To address potential bias arising from this sample imbalance, propensity score matching (PSM) was employed to construct a comparable control group, and balance tests confirmed that baseline differences were substantially reduced after matching (see Results section and Table S1).

### Measurements

#### Dependent Variables

*Health of older adults*. In this study, the health of older adults was assessed using three dimensions: self-rated health, physical health, and mental health. Self-rated health provides a comprehensive evaluation of the health status of older adults, taking into account factors such as family genetic history, past disease history, and social adaptability. It is measured by asking the question, “How do you rate your health at present?” with response options ranging from “very bad” to “very good.” The responses are assigned values of 1 to 5, respectively, with higher scores indicating better self-rated health.

Physical health in this study was assessed based on the question, “ Have you felt not well in the past two weeks?” Respondents were assigned a value of “0” for “yes” and “1” for “no,” indicating whether they felt unwell during that period.

This study measured the mental health of older adults in both positive and negative ways. Positive questions included, “Do you always think well no matter what is happening to you?” “Do you like to keep things clean and tidy?” “Are you in charge of your own affairs?” Negative measurement questions include, “Are you nervous or afraid?”, “Do you feel lonely?” “Do you feel that the older you get, the less useful you are, and that you have a hard time doing everything?” The negatively measured questions were reversed and assigned values. The larger the value of the positive measurement question, the smaller the value of the negative mental health. Each question was scored from 1 to 5, and the total score for the six questions was calculated, with larger values indicating better mental health in older adults [24].

#### Independent variables

*Community-based integrated care*. In China, community-based integrated care for older adults is typically delivered through community health service centres, neighbourhood committees, and contracted social organizations, primarily via home-based and on-site community services [5]. These services aim to address the multidimensional needs of older adults by integrating resources across health, social, and family domains.

Drawing on the CLHLS questionnaire, the community-based integrated care variable in this study was constructed based on the availability of four types of services in the respondent’s community: (1) daily care, including personal care and daily shopping assistance; (2) medical services, including home visits by doctors and medicine delivery; (3) health services, including the provision of health knowledge and education; and (4) psychological support, including emotional comfort, companionship, and the organization of social and recreational activities. These four components collectively represent an integrated service model that combines living assistance, health management, and psychosocial support. The community-based integrated care variable was assigned a value of 1 if the respondent’s community provides all four types of services, and 0 otherwise.

#### Control Variables

Various factors, including individuals’ inherent demographic attributes, socioeconomic status, and health conditions, influence the well-being of older adults [25]. In this study, control variables were identified based on existing literature. These variables encompass several aspects, including demographic characteristics such as age, gender, education, and household registration. Additionally, family characteristics such as marital status, property ownership, residential arrangement, and number of children were considered.

The study also took into account social security characteristics, including public health insurance, public pension insurance, commercial health insurance, and commercial pension insurance. Furthermore, health characteristics such as the number of chronic diseases and body mass index (BMI) were included as control variables. The study employed the classification of BMI according to the People’s Republic of China Health Industry Standard WS/T428-2013 Adult Weight Determination. BMI categories used in the study included underweight (BMI < 18.5), normal weight (18.5 ≤ BMI < 24.0), overweight (24.0 ≤ BMI < 28.0), and obesity (BMI ≥ 28.0).

### Analytical strategies

This study utilized OLS to test whether community-based integrated care would have a significant impact on the health of older adults, i.e:


1 $${\text{Health}}_{i}=\alpha_{1}+\beta_{1}{\text{Care}}_{i}+\gamma_{1}M_{i}+\varepsilon_{1i}$$

where *Care*_*i*_ is whether the ith older person receives community-based integrated care (*Care*_*i*_ = 0 for not receiving; *Care*_*i*_ = 1 for acceptance); *Health*_*i*_ is the health status of the ith older person; *M*_*i*_ is the matrix of covariates affecting the health of the older person; ɛ_1__*i*_ is the randomized disturbance term.

The PSM approach was used in this study to account for selection bias. When determining and assessing the effect of community-based integrated care on older individuals’ health, endogeneity—which can result from biased sample selection and bidirectional causality—must be taken into account. PSM is frequently applied to mitigate bias in sample selection. By concentrating on the average treatment effect (ATT), we aimed to ascertain the influence of community-based integrated care services on the health outcomes of senior citizens. The PSM estimates are presented below:


2 $$\begin{array}{c} {\text{ATT}}_{\text{PSM}}=E\left[{\text{Health}}_{1\text{i}}|{\text{Care}}_{i}=1,p(X_{i})\right]\\ \;-E\left[{\text{Health}}_{0\text{i}}|{\text{Care}}_{i}=0,p(X_{i})\right] \end{array}$$

where Health_1_ and Health_0_ represent the health outcomes for the treatment and control groups, respectively.

Endogeneity due to bi-directional causality can be addressed through an instrumental variable (IV) strategy, and our study used the rate of community-based integrated care service provision as an instrumental variable for individual community-based integrated care. The two-stage least squares (2SLS) method was used for estimation, with Equation (3) as the first-stage estimating equation, where the dependent variable is the receipt of community-based integrated care services by the older people, and the main explanatory variable is the rate of provision of community-based integrated care at the provincial level; and Equation (4) as the second-stage estimating equation, where the dependent variable is the health level of the elderly, and the main independent variable is the fitted value of community-based integrated care.


3 $${\text{Care}}_{i}=\alpha_{2}+\beta_{2}\text{Pro\_Care}+\gamma_{2}M_{i}+\varepsilon_{2i}$$

4 $${\text{Health}}_{i}=\alpha_{3}+\beta_{3}\hat{{\text{Care}}_{i}}+\gamma_{3}M_{i}+\varepsilon_{3i}$$

## Results

### Descriptive statistical analysis

Table 1 presents the description and results of the descriptive statistics for the relevant variables. Among the respondents, 396 individuals received community-based integrated care, while 9602 individuals did not receive such services. The respondents consisted of 40.3% from the lower age group and 59.7% from the higher age group. In terms of gender distribution, 46.5% of the respondents were male, while 53.5% were female. Regarding the means of the variables, older individuals who received community-based integrated care reported higher levels of self-rated health (3.732 vs. 3.448), physical health (0.864 vs. 0.849), and mental health (25.172 vs. 23.139) compared to those who did not receive such care. The differences in self-rated health and mental health between the two groups were statistically significant, whereas the difference in physical health was not.

**Table 1:** Definition of variables, assignment of values and results of their descriptive statistics.


VARIABLES		FULL SAMPLE (N = 9998)		CONTROL GROUP (N = 9602)		TREATMENT GROUP (N = 396)		T-test
		
		MEAN	S. D	MEAN	S. D	MEAN	S. D	

Older people Health	Self-rated health	3.459	0.888	3.448	0.888	3.732	0.853	–0.284***

	Physical health	0.850	0.357	0.849	0.358	0.864	0.344	–0.015

	Mental health	23.220	3.342	23.139	3.325	25.172	3.163	–2.033***

Demography Characteristics	Age							

	Age < 80 (1 = yes; 0 = no)	0.403	0.491	0.402	0.490	0.434	0.496	–0.032

	Age >= 80 (1 = yes; 0 = no)	0.597	0.491	0.598	0.490	0.566	0.496	0.032

	Gender (1 = male; 0 = female)	0.465	0.499	0.464	0.499	0.490	0.501	–0.026

	Education							

	Illiterate (1 = yes; 0 = no)	0.378	0.485	0.382	0.486	0.288	0.453	0.094***

	Primary (1 = yes; 0 = no)	0.295	0.456	0.296	0.457	0.258	0.438	0.039*

	Junior high school and above (1 = yes; 0 = no)	0.327	0.469	0.322	0.467	0.455	0.499	–0.133***

	Household registration (1 = urban; 0 = rural)	0.280	0.449	0.276	0.447	0.381	0.486	–0.105***

Family Characteristics	Marital status (1 = married; 0 = unmarried)	0.457	0.498	0.456	0.498	0.490	0.501	–0.034

	Property ownership	0.930	0.255	0.930	0.256	0.942	0.234	–0.012

	Residential arrangement (1 = not living alone; 0 = living alone)	0.831	0.375	0.833	0.373	0.793	0.406	0.040**

	Number of children	3.533	1.703	3.557	1.706	2.960	1.514	0.597***

Social security characteristics	Public medical insurance	0.886	0.318	0.889	0.314	0.811	0.392	0.078***

	Public pension insurance	0.272	0.445	0.275	0.447	0.187	0.390	0.088***

	Commercial medical insurance	0.007	0.082	0.007	0.082	0.005	0.071	0.002

	Commercial pension insurance	0.014	0.117	0.014	0.117	0.013	0.112	0.001

Health characteristics	Chronic disease							

	No chronic disease (1 = yes; 0 = no)	0.294	.456	.295	.456	.275	0.447	0.020

	Single chronic disease (1 = yes; 0 = no)	0.308	.462	.307	.461	.331	0.471	–0.024

	Multiple chronic diseases (1 = yes; 0 = no)	0.398	.489	.398	.489	0.394	0.489	0.004

	BMI							

	Underweight (1 = yes; 0 = no)	0.143	0.350	0.145	0.352	0.086	0.281	0.059***

	Normal weight (1 = yes; 0 = no)	0.498	0.500	0.500	0.500	0.465	0.499	0.035

	Overweight (1 = yes; 0 = no)	0.242	0.428	0.240	0.427	0.288	0.453	–0.048**

	Obesity (1 = yes; 0 = no)	0.117	0.321	0.115	0.319	0.162	0.369	–0.047***

Lifestyle characteristics	Smoking (1 = yes; 0 = no)	0.162	0.368	0.163	0.369	0.139	0.346	0.024

	Drinking alcohol (1 = yes; 0 = no)	0.157	0.364	0.155	0.362	0.194	0.396	–0.039**

	Exercise (1 = Yes; 0 = No)	0.332	0.471	0.330	0.470	0.384	0.487	–0.054**


*Note*: *, **, and *** indicate 10%, 5%, and 1% significance, respectively; same below. Tests of difference were analyzed using t-tests.

### Analysis of baseline results

Columns (1) to (3) in Table 2 show the results of the OLS estimation of the influence of receiving versus not receiving community-based integrated care on the health of older individuals. Older adults who received community-based integrated care services showed significantly higher levels of self-rated health (β = 0.529, p < 0.01) and mental health (β = 1.065, p < 0.01), suggesting a positive association between the intervention and health outcomes. In contrast, its impact on physical health was not statistically significant.

**Table 2:** Estimates of the impact of community-based integrated care services and their level of integration on the health of older people.


	VARIABLES	(1)	(2)	(3)
		
		SELF-RATED HEALTH	PHYSICAL HEALTH	MENTAL HEALTH

	Community-based integrated care	0.529*** (0.096)	0.089 (0.155)	1.065*** (0.098)

Demographic characteristics	Age ≥ 80	0.037 (0.046)	–0.004 (0.073)	–0.077* (0.043)

	Gender	0.027 (0.045)	0.113 (0.069)	–0.044 (0.042)

	Primary	0.075 (0.050)	0.037 (0.077)	0.292*** (0.047)

	Junior high school and above	0.040 (0.051)	0.098 (0.080)	0.312*** (0.048)

	Household registration	0.177*** (0.048)	0.023 (0.071)	0.430*** (0.045)

Family characteristics	Marital status	–0.136*** (0.047)	0.030 (0.072)	0.299*** (0.044)

	Property ownership	0.148* (0.079)	–0.030 (0.112)	0.183** (0.072)

	Residential arrangement	0.117** (0.055)	0.056 (0.081)	0.078 (0.053)

	Number of children	0.016 (0.012)	–0.007 (0.019)	–0.004 (0.011)

Social security characteristics	Public medical insurance	–0.066 (0.062)	0.068 (0.090)	–0.077 (0.058)

	Public pension insurance	0.007 (0.043)	0.001 (0.065)	0.098** (0.040)

	Commercial medical insurance	0.062 (0.231)	–0.495 (0.329)	0.048 (0.203)

	Commercial pension insurance	0.310* (0.187)	–0.156 (0.239)	0.222 (0.146)

Health characteristics	Single chronic disease	–0.425*** (0.046)	–0.604*** (0.089)	–0.065 (0.044)

	Multiple chronic diseases	–1.113*** (0.048)	–1.289*** (0.081)	–0.325*** (0.044)

	Underweight	–0.312*** (0.057)	–0.458*** (0.084)	–0.229*** (0.053)

	Overweight	0.147*** (0.047)	0.010 (0.074)	0.144*** (0.044)

	Obesity	–0.005 (0.064)	–0.295*** (0.088)	0.008 (0.060)

Lifestyle characteristics	Smoking	0.043 (0.055)	0.047 (0.092)	0.087* (0.051)

	Drinking	0.345*** (0.054)	0.190** (0.095)	0.333*** (0.051)

	Exercise	0.563*** (0.042)	0.079 (0.065)	0.645*** (0.039)

Pseudo R^2^		0.0355	0.0452	0.0231

N		9998	9998	9998


*Note*: Standard errors in parentheses; ***p < 0.01, **p < 0.05, *p < 0.1.

### Results and testing of PSM analyses

This study used balance tests and predicted the fitted values of conditional probabilities of older individuals’ access to community-based integrated care services using logit models, prior to the PSM analysis. Table S1 and Figure S1 in the supplementary file display the results.

Table 3 shows the estimation results of the influence of community-based integrated care on the health of older individuals, as obtained using the PSM approach. The estimates produced through several matching methods consistently reflect the influence of community-based integrated care on the health of older persons, confirming the robustness of the results [26]. Community-based integrated care was positively associated with self-rated health among older adults, with average treatment effect on the ATT values ranging from 0.253 to 0.296, all significant at the 1% level. However, no statistically significant effects were observed on physical health. In contrast, the intervention was significantly and positively associated with mental health, with ATT estimates ranging from 1.777 to 1.935, also significant at the 1% level, indicating a pronounced positive impact on the psychological well-being of older adults.

**Table 3:** PSM results of community-based integrated care on the health of older adults.


	(1)	(2)	(3)	(4)

Panel 1: self-rated health				

T	3.731	3.731	3.731	3.732

C	3.454	3.478	3.460	3.436

Difference	0.277*** (0.050)	0.253*** (0.044)	0.271*** (0.044)	0.296*** (0.048)

Panel 2: physical health				

T	0.863	0.863	0.863	0.864

C	0.846	0.853	0.852	0.847

Difference	0.017 (0.020)	0.010 (0.018)	0.011 (0.018)	0.017 (0.020)

Panel 3: mental health				

T	25.173	25.173	25.173	25.172

C	23.395	23.386	23.237	23.294

Difference	1.777*** (0.186)	1.787*** (0.165)	1.935*** (0.163)	1.878*** (0.174)


*Note*: *p < 0.10, ***p < 0.01, **p < 0.05. Column (1) shows estimates by k-nearest neighbor matching; Column (2) shows estimates by radius matching; Column (3) shows estimates by kernel matching; Column (4) shows estimates by Mahalanobis matching.

### Endogeneity test

To deal with the selectivity bias caused by unobservable variables and potential reciprocal causality between integrated community care and older adults’ health, we used an IV approach with the rate of integrated community care provision in the provinces where older adults live as an instrumental variable. The first stage of the IV test revealed a significant positive association (with a statistical significance level of 1%) between the provision of integrated care services in communities where older adults live and the instrumental variable. This finding supports the validity of the instrumental variable.

Table 4 shows that community-based integrated care was significantly and positively associated with self-rated health (β = 2.894, p < 0.01), physical health (β = 0.267, p < 0.01), and mental health (β = 14.911, p < 0.01) among older adults. The consistency between the estimates obtained from the IV method and those from the PSM approach reinforces the robustness of the findings, particularly with respect to the positive effects on self-rated and mental health. Moreover, the IV estimates reveal a statistically significant positive effect on physical health, in contrast to the non-significant association identified through the PSM analysis. This discrepancy highlights the importance of addressing potential endogeneity and accounting for unobserved confounding factors.

**Table 4:** The 2SLS results of community-based integrated care on old people’s health.


	VARIABLES	(1)	(2)	(3)	(4)
			
		COMMUNITY-BASED INTEGRATED CARE	SELF-RATED HEALTH	PHYSICAL HEALTH	MENTAL HEALTH

	Community-based integrated care ratio	0.893*** (0.045)			

	Community-based integrated care		2.894*** (0.302)	0.267*** (0.093)	14.911*** (1.279)

Demographic characteristics	Age ≥ 80	0.003 (0.005)	–0.006 (0.025)	–0.002 (0.009)	–0.232** (0.101)

	Gender	0.001 (0.005)	0.014 (0.024)	0.014* (0.009)	–0.044 (0.098)

	Middle school	0.001 (0.005)	0.033 (0.026)	0.004 (0.010)	0.507*** (0.108)

	High school and above	0.016*** (0.005)	–0.028 (0.027)	0.007 (0.010)	0.333*** (0.111)

	Household registration	–0.004 (0.005)	0.070*** (0.026)	0.002 (0.009)	0.654*** (0.108)

Family characteristics	Marital status	0.001 (0.005)	–0.070*** (0.025)	0.003 (0.009)	0.471*** (0.101)

	Property ownership	0.020** (0.008)	0.028 (0.041)	–0.009 (0.015)	0.119 (0.165)

	Residential arrangement	–0.011** (0.006)	0.093*** (0.030)	0.012 (0.010)	0.347*** (0.121)

	Number of children	–0.005*** (0.001)	0.025*** (0.006)	0.001 (0.002)	0.094*** (0.026)

Social security characteristics	Public medical insurance	–0.022*** (0.006)	0.042 (0.035)	0.016 (0.012)	0.218 (0.149)

	Public pension insurance	–0.010** (0.004)	0.035 (0.022)	0.003 (0.008)	0.309*** (0.092)

	Commercial medical insurance	–0.015 (0.024)	0.072 (0.114)	–0.064 (0.051)	0.299 (0.515)

	Commercial pension insurance	–0.019 (0.016)	0.194** (0.090)	–0.014 (0.033)	0.607* (0.365)

Health characteristics	Single chronic disease	0.002 (0.005)	–0.199*** (0.025)	–0.056*** (0.008)	–0.174* (0.103)

	Multiple chronic diseases	–0.007 (0.005)	–0.493*** (0.025)	–0.150*** (0.009)	–0.583*** (0.102)

	Underweight	–0.005 (0.006)	–0.120*** (0.029)	–0.055*** (0.011)	–0.291** (0.117)

	Overweight	–0.001 (0.005)	0.049* (0.025)	0.000 (0.009)	0.155 (0.105)

	Obesity	0.008 (0.006)	–0.058 (0.035)	–0.044*** (0.013)	–0.208 (0.145)

Lifestyle characteristics	Smoking	–0.011* (0.006)	0.048 (0.029)	0.007 (0.010)	0.302*** (0.117)

	Drinking	0.010* (0.006)	0.125*** (0.030)	0.017* (0.010)	0.418*** (0.124)

	Exercise	0.004 (0.004)	0.243*** (0.022)	0.009 (0.008)	1.110*** (0.092)

R^2^		0.051	0.923	0.852	0.970

N		9998	9998	9998	9998


*Note*: Standard errors in parentheses; ***p < 0.01, **p < 0.05, *p < 0.1.

### Heterogeneity test

Table 5 displays the findings of the heterogeneity examination across age, gender, residential arrangement, and household registration for older people. Receiving community-based integrated care can lead to greater physical and mental health advantages for older persons aged 80 and over, female older adults, older adults living alone, and older adults living in rural areas. The level of integration of community-based integrated care has a greater impact on improving physical and mental health with increased community-based integrated care for older adults aged 80 years and older, female older adults, older adults living alone, and older adults living in rural areas.

**Table 5:** Results of the Heterogeneity Analysis.


	COMMUNITY-BASED INTEGRATED CARE		

	SELF-RATED HEALTH	PHYSICAL HEALTH	MENTAL HEALTH

Panel 1: By age			

Age < 80 (N = 4033)	0.496*** (0.154)	–0.118 (0.236)	1.068*** (0.148)

Age ≥ 80 (N = 5965)	0.639*** (0.128)	0.314 (0.214)	1.101*** (0.134)

Panel 2: By gender			

Male (N = 4650)	0.334** (0.150)	–0.045 (0.218)	0.908*** (0.136)

Female (N = 5348)	0.763*** (0.143)	0.333 (0.222)	1.191*** (0.139)

Panel 3: By residential arrangement			

Alone (N = 1688)	0.691*** (0.222)	1.071** (0.479)	1.358*** (0.192)

Not alone (N = 8310)	0.493*** (0.107)	–0.027 (0.167)	0.979*** (0.112)

Panel 4: By household registration			

Rural (N = 7196)	0.621*** (0.119)	0.098 (0.221)	1.496*** (0.123)

Urban (N = 2802)	0.457*** (0.165)	0.823 (0.619)	0.457*** (0.152)


*Note*: Robust standard errors in parentheses. ***p < 0.01, **p < 0.05, *p < 0.1.

## Discussion

The most significant conclusion of this study is that community-based integrated care is positively associated with the physical and mental health of older persons. To begin, community-based integrated care includes the medical and nursing support that older people require, such as frequent health screenings, medication management, and rehabilitation services. This may aid in the early detection and management of chronic diseases [27], potentially reducing the risk of complications and contributing to better physical function [28]. Secondly, community-based integrated care offers psychological support, social connection, and recreational activities to help older persons maintain their mental health and participate in society [29,30]. Community-based integrated care, which provides services such as home care and aid with daily living, may help older people address daily living challenges, and is also associated with greater self-esteem and confidence. This favorable impact contributes to the physical and emotional wellbeing of older people [31,32]. Finally, community-based integrated care provides older persons a social support system. Organizing and encouraging older persons to participate in community events minimizes the chances of loneliness and depression while increasing their sense of well-being and life satisfaction [33,34].

These findings are broadly consistent with international evidence on integrated care for older adults. Studies conducted in European contexts have similarly reported positive associations between integrated community care and mental health outcomes among older populations [35,36]. However, a notable distinction in the Chinese context is that community-based integrated care operates within a policy framework that emphasizes “aging in place” as the primary model of elderly care, with communities serving as the central platform for resource integration. Unlike many Western countries where integrated care is primarily coordinated through primary healthcare systems, China’s model relies more heavily on the collaboration between community health service centres, neighbourhood committees, and social organizations. This institutional arrangement may partially explain why the observed associations are particularly strong for mental health: the community-based delivery model inherently provides social interaction and a sense of belonging, which are key determinants of psychological well-being among older adults.

From a theoretical perspective, social support theory offers a useful lens for interpreting these findings. According to Cohen and Wills [37], social support can buffer the negative effects of stress on health through both direct and indirect pathways. Community-based integrated care, by simultaneously providing instrumental support (daily care, medical services), informational support (health knowledge), and emotional support (psychological comfort and social activities), may activate multiple protective pathways. This multi-dimensional support structure may be particularly beneficial for mental health, as it addresses not only physical health needs but also the psychosocial factors that are strongly associated with depression and anxiety among older adults, including loneliness, perceived helplessness, and social isolation.

The findings emphasize the importance of integrated community care. The traditional single-care approach is no longer adequate for older individuals’ various care needs [3,38]. Older adults have diverse and complex needs for long-term care services, which include medical care services such as medication distribution, pain management, and rehabilitation training [39]. Community-based integrated care, by combining multiple nursing resources and services, can provide more complete, integrated, and efficient nursing assistance for older individuals’ self-health and mental health.

It is also worth noting that the non-significant association between community-based integrated care and physical health in the OLS and PSM analyses, compared to the significant association found in the IV analysis, warrants further discussion. One possible explanation is that the relationship between care services and physical health may be more susceptible to confounding by health-related selection effects: older adults in poorer physical condition may be more likely to both reside in communities with care services and report lower physical health, thereby attenuating the observed association. The IV approach, by addressing such endogeneity, may more accurately capture the underlying relationship. This finding underscores the methodological importance of accounting for unobserved confounders in observational studies of care interventions.

Further analysis indicated disparities in the effects of community-based integrated care and its level of integration on the physical and mental health of older individuals across subgroups. In terms of age, community-based integrated care and its level of integration were more effective at improving the health of older adults than younger adults. It has been discovered that older people require more care than the younger individuals [40]. On the one hand, older persons have an increased risk of numerous chronic diseases with incurable and multiple complications [41], indicating a greater need for an integrated and comprehensive treatment paradigm than younger older adults. On the other hand, mobility limitations among older people make it difficult for them to visit hospitals, so there is a greater demand for home-based medical care services [42]. As a result, community-based integrated care can assist older persons in improving their health management and overall physical and mental health.

In terms of gender, community-based integrated care and its level of integration have greater effects on the health outcomes of older women. One possible explanation is that, as a result of the traditional gender division of labour, male older adults tend to have a higher social status and more economic resources that can be exchanged for their children, giving them a greater say and initiative in obtaining intergenerational care from their children. They have more influence and initiative in seeking intergenerational care help from their children, therefore they are less likely to require community care services. Despite the fact that female older adults, as the “main family,” devote more energy, labor, and time to family life and production activities, they are at a distinct disadvantage in terms of obtaining intergenerational care support from their children, and thus their health status is more likely to be impacted by community-based integrated care. Additionally, women’s longer life expectancy is likely another contributing factor. Older women are more likely to outlive their spouses, which reduces their access to spousal caregiving and increases their dependence on external care sources, including community-based services [6]. The combination of socioeconomic disadvantage and greater likelihood of widowhood may thus make older women particularly responsive to the availability of community-based integrated care.

For older adults living alone, the benefits of community-based integrated care were more noticeable when it came to their mental and physical health while they were in residential settings. This is in line with other studies that highlighted the heightened susceptibility and medical requirements of single individuals [43]. In terms of financial support, life care, and emotional comfort, older adults living alone typically do not receive the kind of long-lasting intergenerational support from their children [44], making the need for community-based integrated care even more pressing. Furthermore, community-based integrated care can successfully compensate for the deficiencies that older individuals living alone suffer in terms of life care, health management, and humanistic care. Research indicates that addressing the internal and external variables that contribute to falls in older adults living alone might reduce the risk of falls and increase the capacity of these individuals to take care of themselves in the event of an incident [45]. Individuals who do not live alone, such as those who live with their families or in institutionalized settings, have access to formal care in institutions or informal care support from their children, which has the effect of “crowding out” community care services, which are then only supplemental to addressing older people’s long-term care needs.

For older persons living in rural areas, community-based integrated care has a higher effect on health outcomes when it comes to household registration. Access to health care services is frequently hampered in rural areas by structural inequities in the allocation of health care resources and a lack of human resources [46]. Due to their relative lack of access to resources, older adults living in rural areas are more likely to have lower expectations of care services and to subjectively see community-based integrated care as providing concrete benefits. In rural locations, the lack of medical services can be compensated for and the health needs of older individuals living in rural areas can be satisfied by offering community-based integrated care services. The demand for community-based integrated care will decline in urban areas because there is a greater abundance of forms and contents of care services available, a more developed and standardized market for long-term care services, and relatively better economic and social security conditions for urban older adults.

The findings of this study carry several policy implications for the development of community-based elderly care in China. First, given the strong positive association between integrated care and health outcomes, policymakers should prioritize expanding the coverage of comprehensive community care services, particularly in rural and economically underdeveloped areas where service provision remains fragmented. The current reality—where only a small fraction of communities provide all four types of care services simultaneously—suggests that substantial investment in service infrastructure and workforce development is needed. Second, the heterogeneity findings point to the need for targeted policy design. The stronger associations observed among older adults aged 80 and above, women, those living alone, and rural residents suggest that these subgroups may benefit most from integrated community care. Policy efforts could focus on developing tailored service packages for these populations, such as enhanced home-visit programmes for the oldest-old and those living alone, and gender-sensitive care models that account for the distinct caregiving trajectories of older women.

It’s also critical to acknowledge the study’s shortcomings. This study only determined whether or not the older persons interviewed had received community-based integrated care; due to constraints in the primary data, we were unable to ascertain the precise frequency of this type of care. Communities will inevitably differ in how frequently they provide community care services, which could have varying effects on older individuals’ health. Furthermore, given that integrated community care in China is now lacking consistent policy requirements and staff training, the quality of services offered by various communities may also differ. Older people have access to a wide range of social and health services, but the degree to which they have an influence will vary. Future research endeavors could contemplate the use of longitudinal data to delve deeper into the distinct effects of the frequency and quality of care provided by community-based integrated care on the health outcomes of older individuals.

## Conclusions

Using data from the CLHLS2018, this study examined the impact of community-based integrated care, including the degree of integration, and the physical and mental health of Chinese older persons. Within the scope of the available data, the findings suggest that receiving community-based integrated care is positively associated with the self-rated health, physical health, and mental health of older adults, though the strength of these associations varied across outcome measures and analytical methods. The results also indicate that a higher degree of integration may be linked to stronger positive associations with health outcomes, although this pattern warrants further validation. The heterogeneity analysis suggested that these associations tended to be more pronounced among the oldest-old, female older adults, older adults living alone, and rural older adults, compared with their younger, male, cohabiting, or urban counterparts. These subgroup patterns should be interpreted with caution given the relatively small treatment group size.

It should also be noted that this study employs a cross-sectional design, and the findings represent associations rather than causal relationships. Although PSM and IV methods were used to mitigate selection bias and endogeneity, unobserved confounders may still influence the results. Future research should adopt longitudinal or quasi-experimental designs to further establish the causal pathways through which community-based integrated care affects the health outcomes of older adults.

## Additional File

The additional file for this article can be found as follows:

10.5334/ijic.9099.s1 Supplementary File. Supplementary Table S1 and Figure S1. Propensity score matching diagnostics, including balance tests and propensity-score distributions before and after matching.

## Data Availability

Publicly available datasets were analyzed in this study. This data can be found at: https://doi.org/10.18170/DVN/WBO7LK.

## Author Information

Qinglin Xu is a lecturer at the School of Economics and Management, Anhui University of Chinese Medicine, with research interests in health economics and healthy aging. Cangcang Jia is a lecturer in the School of Health Policy and Management of Nanjing Medical University, with research interests in long-term care system, healthy aging and medical and health system reform.
