# Supplementary material for: The Influence of Community-Based Integrated Care on the Physical and Mental Health of Older Adults: Evidence from China
Source: Int J Integr Care. 2026 Sep 30;26(3):21. doi: 10.5334/ijic.9099 (PMC13635667; doi:10.5334/ijic.9099)
Supplement: Supplementary file 1 [file ijic-26-3-9099-s1.pdf]

Supplementary

The logit model results indicate that, at least at the 10% statistical level, residence arrangement, number of children, public medical insurance, public pension insurance, and underweight have a significant negative influence on older adults' access to community-based integrated care services. Age, high school and above, property ownership, obesity, and drinking, have a significant positive effect.

Table S1. Balancing tests after propensity score matching

Variables	Logit Score	Match State of affairs	Mean		Deviation rate (%)	Deviation reduction ratio (%)	Double t-test	
			T	C			T	P>|t|
Age≥ 80	0.242* (0.127)	U	0.566	0.598	-6.5	68.5	-1.28	0.200
		M	0.563	0.574	-2.1		-0.29	0.774
Gender	-0.045 (0.122)	U	0.490	0.464	5.2	-86.7	1.01	0.313
		M	0.490	0.442	9.7		1.36	0.175
Middle school	0.073 (0.149)	U	0.258	0.296	-8.6	54.0	-1.65	0.099
		M	0.259	0.241	4.0		0.58	0.565
High school And above	0.422*** (0.141)	U	0.455	0.322	27.4	84.7	5.52	0.000
		M	0.452	0.472	-4.2		-0.57	0.568
Household registration	0.116 (0.123)	U	0.381	0.276	22.5	73.5	4.57	0.000
		M	0.378	0.350	6.0		0.81	0.416
Marital status	0.137 (0.135)	U	0.490	0.456	6.8	-5.0	1.32	0.185
		M	0.492	0.528	-7.1		-1.00	0.319
Property ownership	0.436* (0.224)	U	0.942	0.930	5.0	-87.0	0.93	0.350
		M	0.942	0.964	-9.3		-1.52	0.130
Residence arrangement	-0.435*** (0.144)	U	0.793	0.833	-10.2	68.1	-2.07	0.038
		M	0.797	0.810	-3.3		-0.45	0.655
Number of children	-0.206*** (0.035)	U	2.960	3.557	-37.0	98.7	-6.85	0.000
		M	2.967	2.975	-0.5		-0.07	0.944
Public medical insurance	-0.552*** (0.137)	U	0.811	0.889	-22.1	83.8	-4.82	0.000
		M	0.812	0.825	-3.6		-0.46	0.644
Public pension	-0.401***	U	0.187	0.275	-21.1	82.8	-3.88	0.000

insurance	(0.133)	M	0.188	0.173	3.6		0.56	0.579
Commercial medical insurance	-0.420 (0.726)	U	0.005	0.007	-2.2	-47.7	-0.41	0.681
		M	0.005	0.008	-3.3		-0.45	0.654
Commercial pension insurance	-0.430 (0.466)	U	0.013	0.014	-1.2	-663.8	-0.22	0.825
		M	0.013	0.003	5.9		1.64	0.101
Single chronic disease	0.082 (0.134)	U	0.331	0.307	5.1	25.0	1.00	0.317
		M	0.330	0.348	-3.8		-0.53	0.599
Multiple chronic diseases	-0.106 (0.133)	U	0.394	0.398	-0.8	-636.6	-0.15	0.880
		M	0.396	0.368	5.7		0.81	0.421
Underweight	-0.412** (0.192)	U	0.086	0.145	-18.7	74.4	-3.31	0.001
		M	0.086	0.102	-4.8		-0.73	0.464
Overweight	0.168 (0.126)	U	0.288	0.240	10.8	57.5	2.17	0.030
		M	0.289	0.269	4.6		0.63	0.526
Obesity	0.416*** (0.152)	U	0.162	0.115	13.6	56.8	2.85	0.004
		M	0.157	0.137	5.9		0.80	0.422
Smoking	-0.251 (0.163)	U	0.139	0.163	-6.6	68.0	-1.26	0.208
		M	0.140	0.132	2.1		0.31	0.755
Drinking	0.285** (0.142)	U	0.194	0.155	10.3	61.1	2.10	0.036
		M	0.193	0.178	4.0		0.55	0.583
Exercise	0.108 (0.113)	U	0.384	0.330	11.2	52.8	2.23	0.026
		M	0.386	0.360	5.3		0.74	0.462

Note: T and C are treatment and control groups, respectively; U and M are before and after matching, respectively.

The balancing test findings show that upon matching, each covariate's standard deviation is substantially lowered to less than 10%. This indicates that individual differences were eliminated when an equilibrium was reached between the group receiving community-based integrated care and the group not receiving such services. The treatment and control samples' kernel density distributions are extremely similar, as shown in Figure S1 in the Supplementary. This suggests that matching significantly lessened sample selection bias and the difference in covariate distributions between the treatment and control samples.

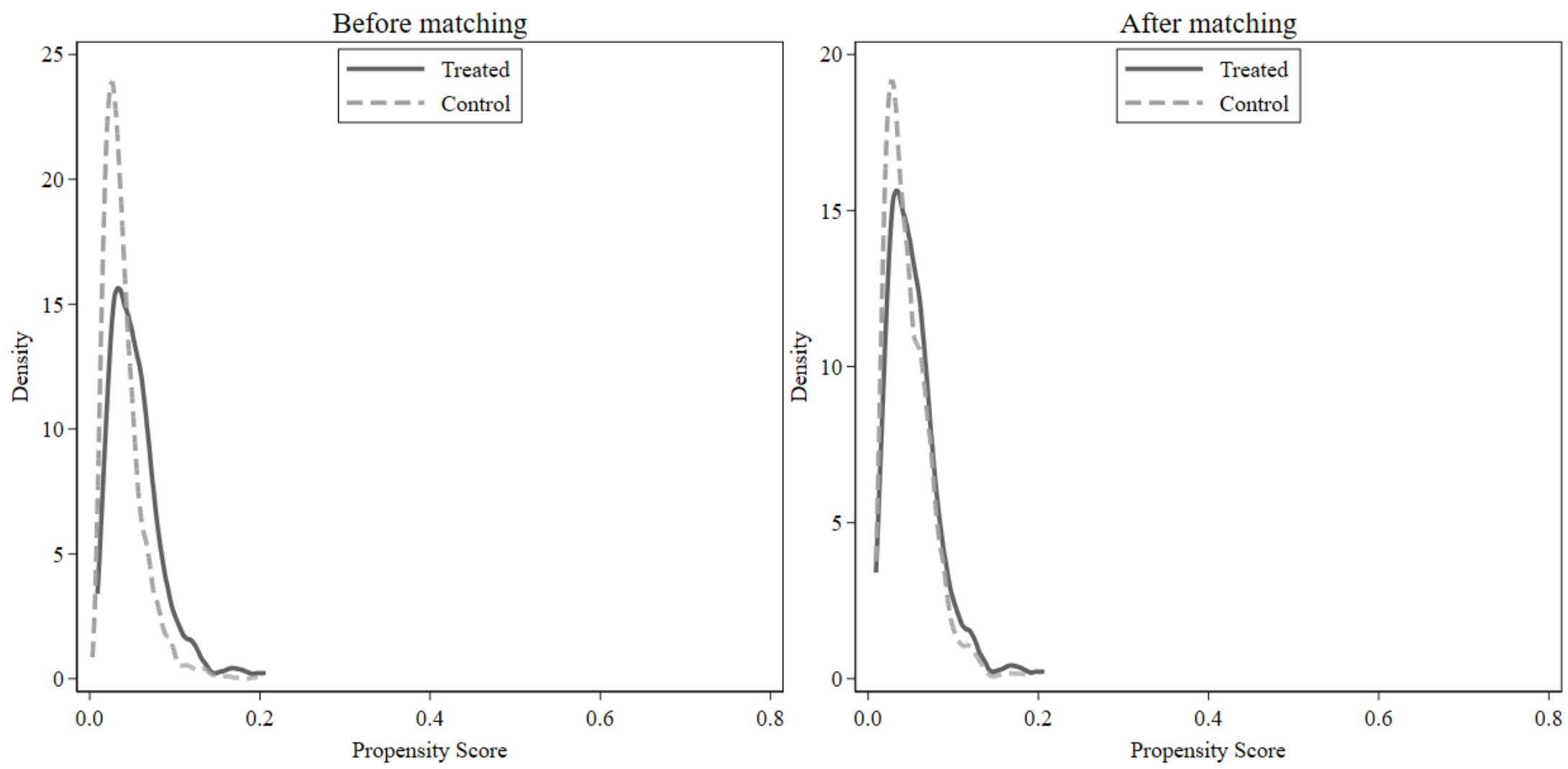

Fig. S1. Kernel density function before (left) and after (right) matching
